# Chitosan-Based Coating Incorporated with Lemon Essential Oil/Rutin Composite Nanoemulsion for Pork Preservation

**DOI:** 10.3390/foods14193351

**Published:** 2025-09-27

**Authors:** Jiaxin Han, Hui Hou, Jiayu Zhu, Xinhui Wang, Fanbing Meng, Weijun Chen

**Affiliations:** Meat Processing Key Laboratory of Sichuan Province, Food Security Publicity and Education Base of Sichuan Province, College of Food and Biological Engineering, Chengdu University, Chengdu 610106, China; 15877423632@163.com (J.H.); houhui8899@126.com (H.H.); jiayuzhu_2024@163.com (J.Z.); wangxinhui@cdu.edu.cn (X.W.); mfb1020@163.com (F.M.)

**Keywords:** lemon essential oil, rutin, chitosan, coating, pork preservation

## Abstract

In this work, a lemon essential oil–rutin composite nanoemulsion was formed and integrated into a chitosan (CS) matrix to form a coating for pork preservation. The introduction of rutin decreased the particle size of the nanoemulsion and suppressed the volatilization of the encapsulated essential oil. The rheological properties of the coating showed that it was a pseudoplastic fluid with shear-thinning behavior, and the apparent viscosity of the system was lower than 0.7 Pa·s. The incorporation of the nanoemulsion significantly (*p* < 0.05) increased the antioxidant and bacteriostatic properties of the CS coating, which was positively correlated with the content of the incorporated nanoemulsion. Pork preservation experiments revealed that the changes in color, the increase in pH, drip loss, thiobarbituric acid-reactive substances, total volatile basic nitrogen and total viable count were significantly (*p* < 0.05) delayed by the coating treatment. These results suggest that the formed lemon essential oil/rutin/CS coating has promising applications in pork preservation.

## 1. Introduction

Pork is one of the most consumed meats in the world. However, the high water activity and high nutrient content of pork make it susceptible to fat oxidation and microbial contamination during storage and transportation, resulting in a rapid deterioration in quality and a significant reduction in shelf-life [1]. This not only poses a food safety hazard to consumers, but also causes considerable economic losses to the meat industry. Therefore, it is crucial to develop safe and efficient preservation technologies to extend the shelf-life and maintain the quality of pork [2].

Refrigeration preservation is one of the most commonly used preservation techniques for pork [3]. However, it can only slow, but not completely inhibit, bacterial growth and biochemical reactions. With the increasing demands for healthy and natural foods, the application of traditional chemical preservatives is limited and has gradually been replaced by natural, nontoxic and nonhazardous preservatives. Among them, bio-preservatives, especially coating preservation technology based on naturally degradable materials, have received widespread attention [4].

Chitosan (CS) is a highly promising coating matrix material because of its excellent film-forming, biocompatible, broad-spectrum antibacterial and antioxidant activities [5]. However, the bactericidal effect of CS alone is not satisfactory. Yu et al. (2025) reported that a CS/agar coating loaded with the bacteriocin phytoalexin FB-2 effectively increased its antimicrobial activity and prolonged the shelf-life of fresh pork [6]. Therefore, the addition of natural active ingredients (e.g., plant essential oils and polyphenolic compounds) to construct a multifunctional composite coating system has become an effective strategy to increase the performance of CS-based materials.

Lemon essential oil (LEO) is rich in terpenoids such as limonene and γ-terpinene, which have strong antimicrobial and antioxidant activities [5]. In meat preservation, LEO could not only inhibit lipid and protein oxidation, but also help to maintain a fresh red color [7]. It was also reported that a low concentration of LEO (1%) enhanced the aroma of tuna meat [8]. However, its application is limited due to its properties of low water solubility, strong odor and low stability [9]. Recently, a number of studies have shown that CS-based films can be used as carriers of LEO in the preservation of meat, fruits and other foods [10,11].

Rutin is a hydrophobic flavonoid with potential health benefits, such as antioxidant, anti-inflammatory and anti-cancer effects [12]. It can synergistically delay lipid oxidation by scavenging reactive oxygen species, chelating metal ions (e.g., Fe^2+^ and Cu^2+^) and inhibiting the activity of the key oxidative enzyme lipoxygenase [13]. Although rutin mainly exists as insoluble crystal particles both in water and oil, its molecules have amphiphilicity and surface activity, and show a significant adsorption trend at the oil–water interface [14]. In the field of food preservation, the integration of rutin into composite coating systems not only provides its own antioxidant advantages but also overcomes the limitations of hydrophobic active ingredients through the carrier-mediated delivery of bioactive compounds [12].

Although the preparation and application of CS-based films activated with LEO have been widely investigated [15], fewer studies focused on CS-based coating. In this work, a LEO/rutin composite nanoemulsion was formed and incorporated into CS to prepare an activated coating, and its preservation effect on pork was systematically evaluated.

## 2. Materials

### 2.1. Materials and Methods

LEO (food grade, limonene content 70–95%), rutin, trichloroacetic acid, thiobarbituric acid, n-butanol, methanol and sodium chloride were supplied by Shanghai Yuanye Bio-Technology Co., Ltd. (Shanghai, China). CS (deacetylation degree ≥ 95%, viscosity 100–200 mPa·s), glycerol and Tween-80 (analytical grade) were obtained from Shanghai Macklin Biochemical Technology Co., Ltd. (Shanghai, China). Fresh pork (lean meat from the hind legs of Neijiang pigs with an initial pH of 5.8–6.2; it was transported to the laboratory within 24 h after slaughter and stored at 4 °C) was purchased from a local market. Agar (analytically pure) was purchased from Shanghai Baiyan Biotechnology Co., Ltd. (Shanghai, China). Yeast extract (analytical grade) was obtained from Beijing Aoboxing Bio-Technology Co., Ltd. (Beijing, China). All chemical reagents used were of analytical grade.

### 2.2. Preparation and Characterization of Lemon Essential Oil/Rutin Composite Nanoemulsion (LEO-NE-R)

#### 2.2.1. Preparation of LEO-NE-R

Rutin (0.05, 0.1, 0.2 and 0.3 mg/mL) was added into 100 mL of 2% (*v*/*v*) Tween-80 aqueous solution and homogenized at 10,000 rpm for 2 min to prepare the aqueous phase by an FJ200-S high-speed shear-dispersing homogenizer (Qwei Instrument Co., Ltd., Hangzhou, China). LEO was mixed with the aqueous phase at a ratio of 5:95 (*v*/*v*) and homogenized at 10,000 rpm for 10 min to obtain a coarse emulsion. It was further treated with a JY92-IIN ultrasonic cell crusher (Xinyi Ultrasonic Equipment Co., Ltd., Ningbo, China) for 8 min (20 °C, 500 W) to obtain LEO-NE-R. According to the concentration of rutin in the aqueous phase, the samples were named as 0.05 LEO-NE-R, 0.1 LEO-NE-R, 0.2 LEO-NE-R and 0.3 LEO-NE-R. The nanoemulsion prepared via the same method without rutin was used as the control and was named LEO-NE.

#### 2.2.2. Particle Size, Zeta Potential (ZP) and Polydispersity Index (PDI) Analysis

After 100-fold dilution with ultrapure water, the average particle size, PDI and ZP of the samples were determined via a Malvern ZEN3600 nanolaser (Malvern, UK)at 25 °C. The scattering angle and equilibrium time were set at 173° and 3 min, respectively.

#### 2.2.3. Transmission Electron Microscopy (TEM) Observation

Samples were dropped on carbon film copper grids, dried under a baking lamp and then negatively stained for 2–3 min with 2% phosphotungstic acid solution. After removal of the excess staining solution and air-drying at room temperature, sample images were randomly acquired at an accelerating voltage of 2–8 kV.

#### 2.2.4. Slow-Release Analysis

Samples (10 g) in glass Petri dishes were moved into an oven (100 °C), and the masses of the samples were measured every two hours [16]. The volatilization rates of LEO were calculated as follows:

(1) $$X\left(\%\right)=\frac{m_{1}-m_{2}}{m_{1}}\times 100$$

where X is the volatilization rate of the sample; m_1_ is the initial sample weight (g); and m_2_ is the sample weight (g) with heating treatment.

### 2.3. Preparation and Characterization of CS-Based Coating Incorporated with LEO-NE-R

#### 2.3.1. Preparation of Coating

The coating was prepared according to the methods of Yang et al. (2019) [17]. Simply, CS (1.5%, *w*/*v*) was dissolved by 1% (*v*/*v*) acetic acid aqueous solution (containing 5% (*w*/*v*) glycerol as plasticization). Then, 1%, 2%, 3% and 4% (*v*/*v*) LEO-NE-R and LEO-NE were added and homogenized for 4 min at 12000 rpm. The groups were named CS-1LEO/NE-R, CS-2LEO/NE-R, CS-3LEO/NE-R, CS-4LEO/NE-R, CS-1LEO/NE, CS-2LEO/NE, CS-3LEO/NE and CS-4LEO/NE based on the content of LEO-NE-R and LEO-NE. The final concentrations of LEO in both experimental and control groups were 0.05%, 0.10%, 0.15% and 0.20% (*v*/*v*), respectively. The rutin concentrations in the experimental groups were 0.00285, 0.00570, 0.00855 and 0.01140 mg/mL, respectively. The blank (CS) group contained neither LEO nor rutin.

#### 2.3.2. Rheological Behavior Analysis

Referring to the method of Xu et al. (2019) with appropriate modifications [18], frequency scans and apparent viscosity measurements were performed at 25 °C with shear rates ranging from 0.1 to 1000 s^−1^.

#### 2.3.3. Antioxidant Activity Analysis

2,2-Diphenyl-1-picrylhydrazyl (DPPH) assay [19]: A mixture of 0.5 mL coating and 2.5 mL DPPH (0.1 mmol/L) ethanol solution was maintained in the dark for 0.5 h, and then its absorbance was recorded at 517 nm (A_1_). The absorbance of the fresh mixture without reaction was A_0_. And the absorbance of the mixture of 0.5 mL coating and 2.5 mL ethanol solution was A_2_. The scavenging rate was computed as follows:

(2) $$DPPH\left(\%\right)=\left(1-\frac{A_{1}-A_{2}}{A_{0}}\right)\times 100$$

2,2′-Azino-bis(3-ethylbenzothiazoline-6-sulfonic acid) (ABTS) assay [20]: A mixture of 4.5 mL ABTS·+ work solution and 0.5 mL coating was reacted at room temperature in the dark for 6 min, and then its absorbance was recorded at 734 nm (A_1_). The absorbance obtained by substituting the coating with hexane or distilled water was A_0_, and the absorbance obtained by replacing the ABTS·+ work solution with ethanol was A_2_. The scavenging rate was computed as follows:

(3) $$ABTS\left(\%\right)=\left(1-\frac{A_{1}-A_{2}}{A_{0}}\right)\times 100$$

#### 2.3.4. Bacteriostatic Activity Analysis

The bacteriostatic activity of the coating was determined via the Oxford cup method [21]. Simply, 100 µL of activated *Staphylococcus aureus* (*S. aureus*), *Listeria monocytogenes* (*L. monocytogenes*) and *Escherichia coli* (*E. coli*) were spread on Luria–Bertani broth solid agar medium. After the surface was dried, an Oxford cup was placed on the surface, and 200 µL coating was added to the Oxford cup. Pure CS was used as a blank control. After incubation at 37 °C for 48 h, the diameter of the inhibition circle was determined via the crisscross method, and the results were averaged from three measurements.

### 2.4. Pork Preservation

#### 2.4.1. Processing of Meat Samples

After removing fascia and fat, fresh pork was cut into small pieces of equal mass, and impregnated with 100 mL coatings for 30 s. After draining, the samples were tightly encapsulated with polyethylene cling film to minimize air inclusion, labeled, placed on trays and stored at 4 ± 0.5 °C. Pork samples without coating treatment were used as the CK.

#### 2.4.2. Color Analysis

Color parameters of the samples were determined using an NH310 handheld colorimeter (3NH Technology Co., Ltd., Guangzhou, China) [22].

#### 2.4.3. pH Analysis

The pH was directly detected by a portable Testo 205 pH meter [23].

#### 2.4.4. Drip Loss Analysis

Before and after the juice on the surface of pork was removed by clean filter paper, the masses were recorded as m_1_ and m_2_, respectively. The drip loss rate was computed as follows:

(4) $$Drip\;Loss\left(\%\right)=\left(\frac{m_{1}-m_{2}}{m_{1}}\right)\times 100$$

#### 2.4.5. Total Volatile Basic Nitrogen (TVB-N) Analysis

This was performed according to the semimicro Kjeldahl method specified in the Chinese National Standard GB 5009.228-2016 [24].

#### 2.4.6. Thiobarbituric Acid-Reactive Substances (TBARS) Analysis

This was measured via the report of Peng et al. (2024) [25]. Five grams of trimmed sample was transferred into a conical flask containing 50 mL trichloroacetic acid solution and shaken for 30 min at 50 °C. The solution was filtered twice after cooling to room temperature. Subsequently, 5 mL filtrate and 5 mL thiobarbituric acid solution were reacted at 90 °C for 40 min. Absorbance was recorded at 532 nm. The TBARS value was computed by a malondialdehyde (MDA) standard curve.

#### 2.4.7. Total Viable Count (TVC) Analysis

Microbial analysis followed the Chinese National Standard GB 4789.2-2016 [26].

### 2.5. Statistical Analysis

Statistical analysis was analyzed via SPSS 27.0 (IBM SPSS Inc., Chicago, IL, USA) with analysis of variance (ANOVA), and statistical significance was defined at *p* < 0.05.

## 3. Results and Discussions

### 3.1. Characterization of LEO-NE-R

#### 3.1.1. Particle Size, PDI and ZP

As shown in Table 1, the average particle sizes of all LEO-NE-R samples were significantly smaller (*p* < 0.05) than the control, suggesting that rutin could enhance the emulsification performance of Tween-80. However, the particle size tended to increase with the increasing addition of rutin. Although rutin mainly exists as insoluble crystal particles both in water and oil, its molecules have amphiphilicity and surface activity, and show a significant adsorption trend at the oil–water interface [14]. Thus, an increase in rutin content may lead to more particles being adsorbed at the interfacial layer of the nanoemulsion, which in turn results in an increase in particle size. Table 1 also shows that the LEO-NE-R samples exhibit smaller PDI values than the control, suggesting that the addition of rutin helps in obtaining nanoemulsions with more uniform droplet distributions [27,28].

#### 3.1.2. TEM Observation

**Figure 1:**
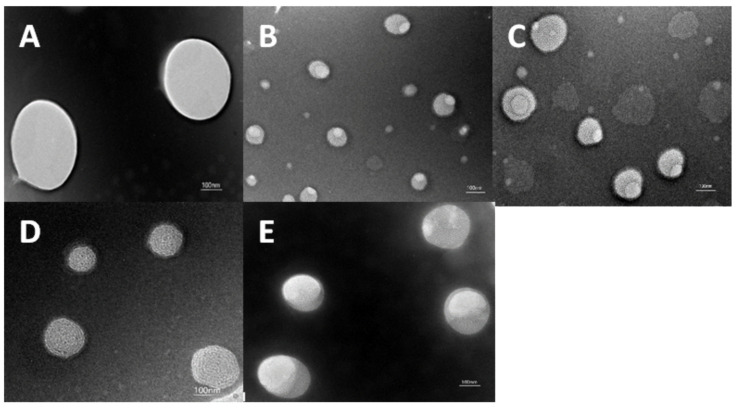
TEM images of different nanoemulsions ((**A**): LEO-NE; (**B**): 0.05 LEO-NE-R; (**C**): 0.1 LEO-NE-R; (**D**): 0.2 LEO-NE-R; (**E**): 0.3 LEO-NE-R).

Figure 1 shows that the nanoemulsion droplets of all the samples exhibit a spherical or ellipsoidal shape with a relatively homogeneous size distribution. However, the droplet sizes observed via TEM were generally smaller than those shown in Table 1. This results from the difference in measurement principles. TEM determines the particle size of droplets in the dehydrated state, whereas Malvern Nano Size determines the hydrated particle size of droplets in the dispersed medium. The latter is determined by the molecular composition inside the droplet and the hydrated ion layer adsorbed on its surface. Therefore, the particle size observed in the dehydrated state is usually smaller than that in the hydrated state [29]. In addition, the control group presents a smooth and homogeneous interface, whereas the LEO-NE-R shows a rough interface. This may be due to the adsorption of rutin particles on the oil–water interfacial layer of the nanoemulsion droplets.

#### 3.1.3. Slow-Release Performance

**Figure 2:**
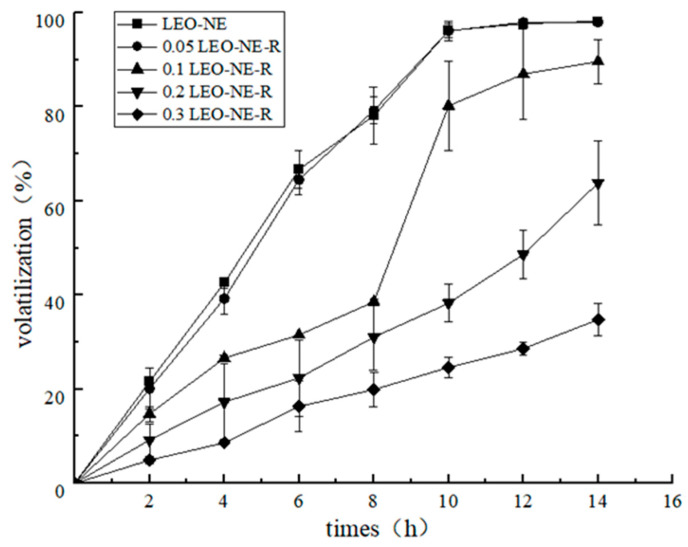
The volatilization rates of different nanoemulsions.

As shown in Figure 2, the volatilization rates of the LEO in all nanoemulsions gradually increased with time, as the high temperature accelerated the volatilization of LEO. Notably, the addition of rutin obviously suppressed the volatilization rate of LEO. With increasing rutin concentration, the volatilization rate of LEO in the LEO-NE-R gradually decreased. This may be because the solid rutin adsorbed at the oil–water interface could increase the mechanical strength of the interfacial layer, thereby enhancing the stability of the composite nanoemulsion [30]. After 14 h of high-temperature treatment, the volatilization rates of each nanoemulsion decreased in the following order: 98.2% (LEO-NE) > 97.9% (0.05 LEO-NE-R) > 89.6% (0.1 LEO-NE-R) > 63.8% (0.2 LEO-NE-R) > 34.7% (0.3 LEO-NE-R). These results revealed that the LEO-NE-R could effectively inhibit the volatilization of essential oils, and the inhibitory effect was positively correlated with the concentration of rutin. Therefore, 0.3 LEO-NE-R was applied to the active CS coating.

It is important to note that the main active constituents of LEO, particularly monoterpenes such as d-limonene, possess an inherent high volatility and are highly prone to oxidative degradation due to their unsaturated chemical structures. The degradation process not only compromises or reduces the bioactivity of the essential oil but also promotes its loss through evaporation [31]. Although the accelerated high-temperature test can reflect the physical stability of the emulsion, it is difficult to accurately predict the chemical stability and true retention rate of monoterpenes (such as limonene) in LEO during long-term cold storage.

### 3.2. Characterization of CS-Based Coating

#### 3.2.1. Rheological Behavior

**Figure 3:**
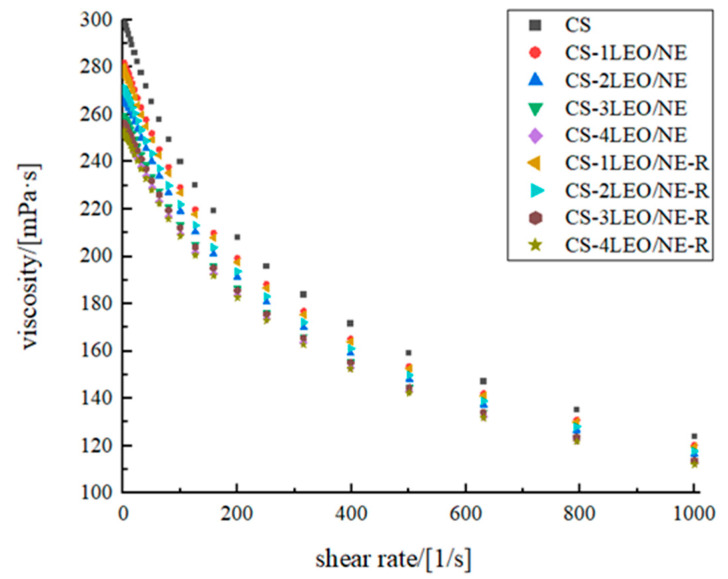
The rheological behavior of different CS-based coatings.

With increasing nanoemulsion concentration, the initial apparent viscosity of the coating gradually decreased (Figure 3), indicating that the introduction of a nanoemulsion may disrupt the intermolecular interactions of CS and enhance fluidity. All coating samples exhibit a continuous decrease in apparent viscosity with increasing shear rate, indicating typical shear-thinning characteristics. This phenomenon confirms that these coatings are all pseudoplastic fluids. When the shear rate increased to 1000 s^−1^, the apparent viscosity of the CS group decreased from 0.3 to 0.124 Pa·s, the CS-1LEO/NE group decreased from 0.281 to 0.120 Pa·s, the CS-2LEO/NE group decreased from 0.266 to 0.116 Pa·s, the CS-3LEO/NE group decreased from 0.259 to 0.113Pa·s, the CS-4LEO/NE decreased group from 0.254 to 0.112 Pa·s, the CS-1LEO/NE-R group decreased from 0.280 to 0.119 Pa·s, the CS-2LEO/NE-R group decreased from 0.270 to 0.117 Pa·s, the CS-3LEO/NE-R group decreased from 0.256 to 0.113 Pa·s and the CS-4LEO/NE-R group decreased from 0.253 to 0.112 Pa·s. These changes were mainly due to the interaction of the CS molecular chains. The molecular chains of CS were untangled and oriented in the flow direction with the increase in shear rate, which weakened the intermolecular interactions and manifested as a decrease in the apparent viscosity [32]. Du et al. (2016) noted that a high-viscosity coating is prone to uneven film distribution due to bubble entrapment, and it is desirable to control the viscosity < 0.7 Pa·s [33]. The viscosities of all studied coatings were lower than this threshold, indicating stable film-forming properties.

#### 3.2.2. Antioxidant Activity

**Figure 4:**
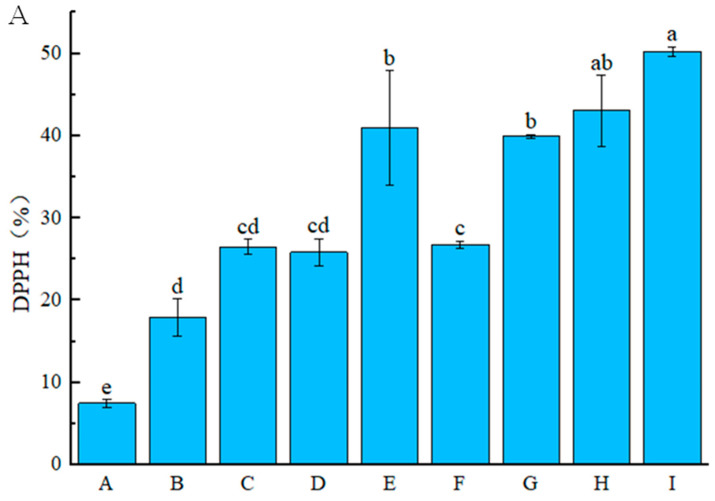

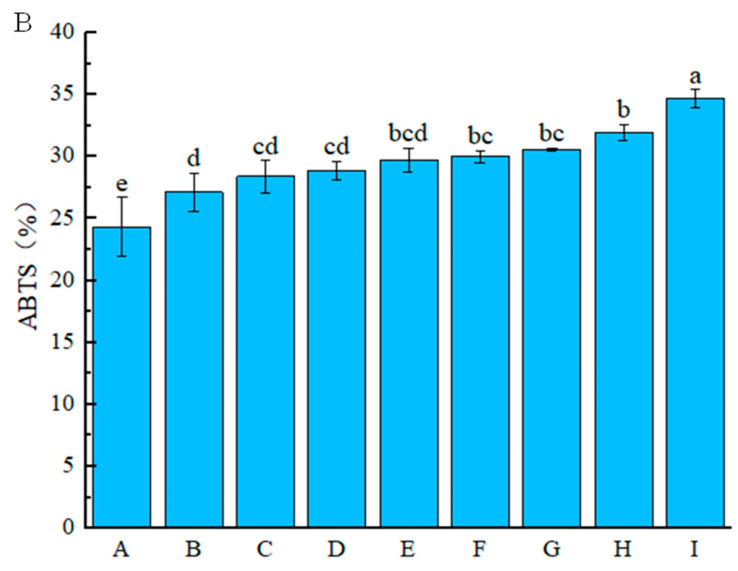
The DPPH radical scavenging ability (**A**) and ABTS radical scavenging ability (**B**) of different CS-based coatings (A: CS, B: CS-1LEO/NE, C: CS-2LEO/NE, D: CS-3LEO/NE, E: CS-4LEO/NE, F: CS-1LEO/NE-R, G: CS-2LEO/NE-R, H: CS-3LEO/NE-R, I: CS-4LEO/NE-R). (Different superscript letters indicate significant difference at *p* < 0.05).

Figure 4A shows that the DPPH radical scavenging rate of CS coating was significantly (*p* < 0.05) lower than other groups. LEO is rich in antioxidant components such as polyphenols and active ester derivatives [34]. Rutin contains more phenolic hydroxyl groups, and these active substances can effectively scavenge DPPH free radicals [35]. Thus, the presence of LEO and rutin significantly enhanced the antioxidant activity of the coating. Notably, the DPPH radical scavenging rates of the CS-LEO/NE-R coatings were significantly (*p* < 0.05) greater than those of the CS-LEO/NE coatings, and increased with the increasing addition of the composite nanoemulsion. This may be attributed to the fact that rutin not only has its own antioxidant capacity but also enhances the slow-release property of the composite nanoemulsion, which may synergistically improve the overall antioxidant performance of the coating. A similar trend was proved by the ABTS assay (Figure 4B). These results indicated that the antioxidant activity of CS-based coatings could be enhanced by the incorporation of CS-LEO/NE-R.

#### 3.2.3. Bacteriostatic Activity

**Figure 5:**
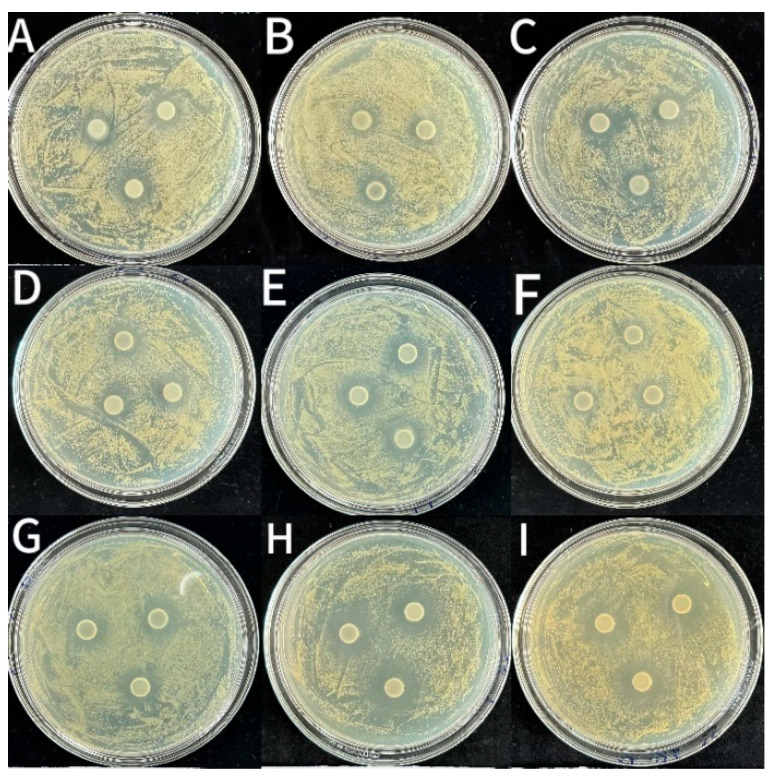
Inhibitory zones of different CS-based coatings against *S. aureus* ((**A**): CS, (**B**): CS-1LEO/NE, (**C**): CS-2LEO/NE, (**D**): CS-3LEO/NE, (**E**): CS-4LEO/NE, (**F**): CS-1LEO/NE-R, (**G**): CS-2LEO/NE-R, (**H**): CS-3LEO/NE-R, (**I**): CS-4LEO/NE-R).

**Figure 6:**
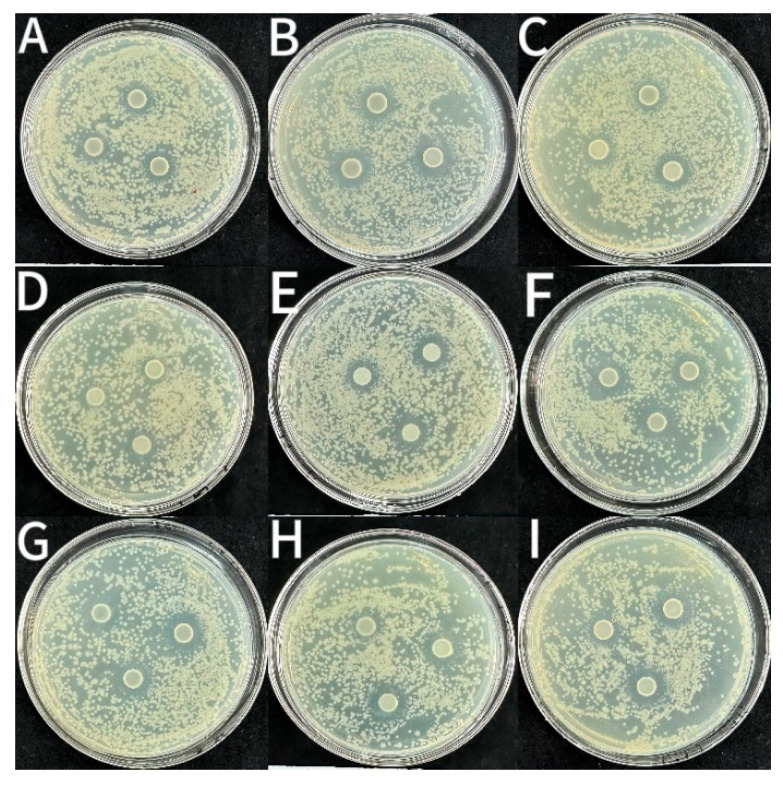
Inhibitory zones of different CS-based coatings against *L. monocytogenes* ((**A**): CS, (**B**): CS-1LEO/NE, (**C**): CS-2LEO/NE, (**D**): CS-3LEO/NE, (**E**): CS-4LEO/NE, (**F**): CS-1LEO/NE-R, (**G**): CS-2LEO/NE-R, (**H**): CS-3LEO/NE-R, (**I**): CS-4LEO/NE-R).

**Figure 7:**
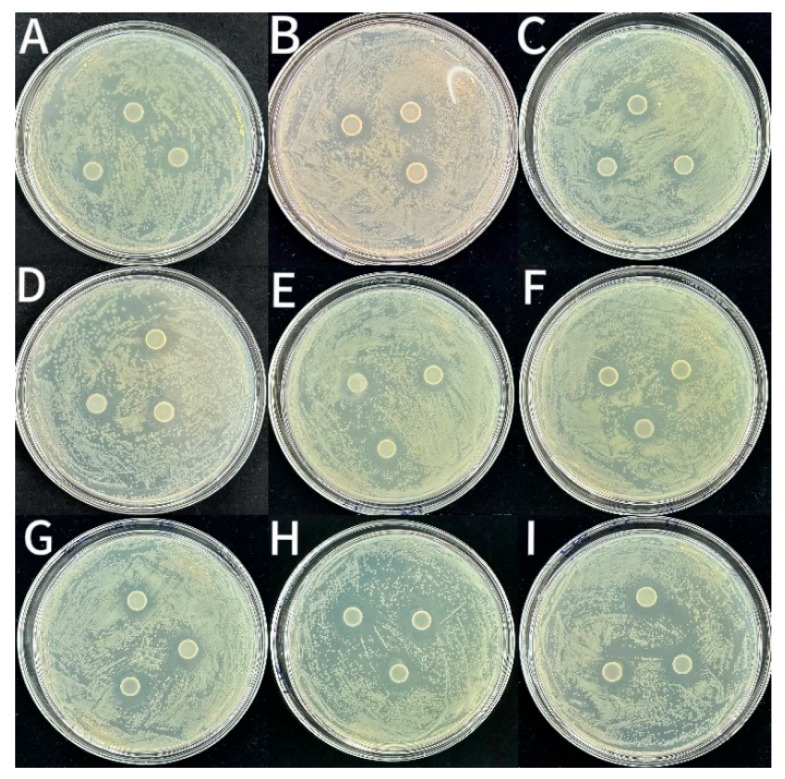
Inhibitory zones of different CS-based coatings against *E. coli* ((**A**): CS, (**B**): CS-1LEO/NE, (**C**): CS-2LEO/NE, (**D**): CS-3LEO/NE, (**E**): CS-4LEO/NE, (**F**): CS-1LEO/NE-R, (**G**): CS-2LEO/NE-R, (**H**): CS-3LEO/NE-R, (**I**): CS-4LEO/NE-R).

As shown in Figure 5, Figure 6 and Figure 7 and Table 2, the CS coating had some bacteriostatic effects on *S. aureus* (14.29 ± 0.2 mm), *L. monocytogenes* (12.88 ± 0.13 mm) and *E. coli* (12.51 ± 0.07 mm). This might be because CS is a cationic polysaccharide with antimicrobial activity. It can disrupt the integrity of the membrane structure via interacting with negatively charged components on the surface of bacterial cell membranes [36]. The diameters of the inhibition circles of coatings incorporated with LEO-NE and LEO-NE-R were significantly (*p* < 0.05) bigger than those of the CS group. Notably, the bacteriostatic activity of coatings incorporated with LEO-NE-R was significantly (*p* < 0.05) stronger than those incorporated with LEO-NE, and increased with increasing incorporation content of LEO-NE-R. This may mainly be due to both LEO and rutin having a bacteriostatic activity [34,35]. The primary active components of LEO (such as limonene) exhibit strong hydrophobicity, enabling them to effectively penetrate and disrupt the phospholipid bilayer of microorganisms [37]. Rutin, as a polyphenolic compound, not only possesses an intrinsic antibacterial activity but also interferes with bacterial energy metabolism and key enzyme activity [12]. In addition, the better slow-release performance of LEO-NE-R may also contribute to this phenomenon [38]. The CS-4LEO/NE-R coating showed the best antioxidant and bacteriostatic activities; thus, it was selected to evaluate its preservation effect for fresh pork.

### 3.3. Pork Preservation

#### 3.3.1. Color

Table 3 shows that the L* of all samples exhibited an initial increase followed by a decrease. The initial rise may be attributed to the denaturation of sarcoplasmic proteins, while the subsequent decline was primarily due to the accumulation of oxidation products from lipids and myoglobin [5]. The initial a* of the CS-4LEO/NE-R group was significantly higher than those of the other groups (*p* < 0.05), which may be associated with a color-enhancing effect contributed by the active components in LEO/NE-R. With prolonged storage, the a* of all groups decreased as a result of the oxidation of myoglobin to metmyoglobin [39]. The CK group showed the most rapid decline in a*, followed by the CS group, the CS-LEO/NE group and finally the CS-LEO/NE-R group. Meanwhile, the b* of all samples showed an increasing trend, which was primarily related to the Maillard reaction induced by lipid oxidation products [40]. Notably, the changes in L*, a* and b* of the CS-4LEO/NE-R group were smaller than those of other groups, suggesting the lowest color deterioration. This may be because the CS-LEO/NE-R treatment significantly delayed myoglobin oxidation owing to its strong antioxidant properties.

#### 3.3.2. pH

**Figure 8:**
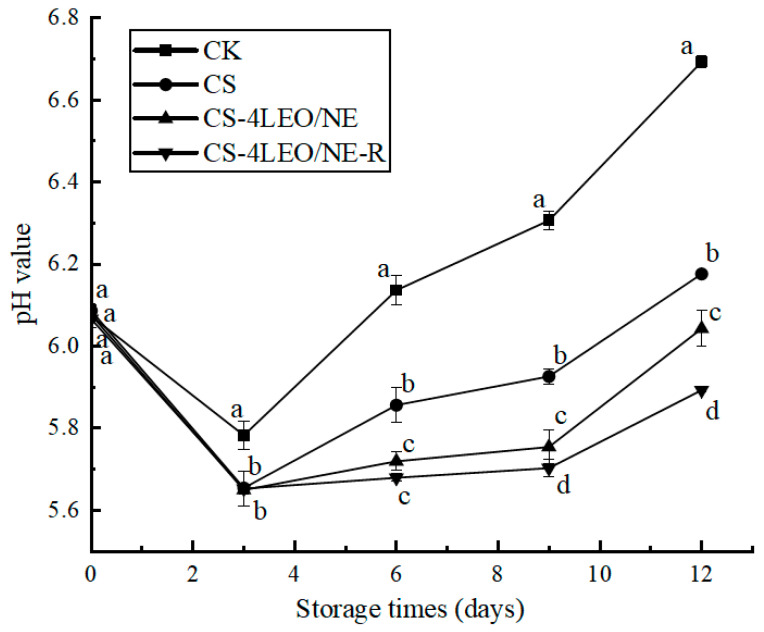
Changes in the pH of pork during different storage periods (Different letters indicate statistically significant differences (*p* < 0.05) at same storage time).

The pH values of the four groups of samples decreased but then increased (Figure 8), which was consistent with the previous research [41,42,43]. The increase in pH may be due to the microbial-induced production of alkaline ammoniacal compounds [44]. The pH values of the CS, CS-4LEO/NE and CS-4LEO/NE-R coating groups were significantly (*p* < 0.05) lower than those of the CK group throughout the storage period, and the CS-4LEO/NE-R group presented the smallest change. On the 12th day, the pH of the CK group rapidly increased to 6.69 ± 0.01, whereas the pH values of the CS, CS-4LEO/NE and CS-4LEO/NE-R groups were 6.18 ± 0.02, 6.04 ± 0.02 and 5.89 ± 0.01, respectively. This indicated that the CS-4LEO/NE-R coating was more effective in limiting the changes in pH of the pork sample, which may be related to its good antimicrobial activity as proved in Figure 5, Figure 6 and Figure 7.

#### 3.3.3. Drip Loss

**Figure 9:**
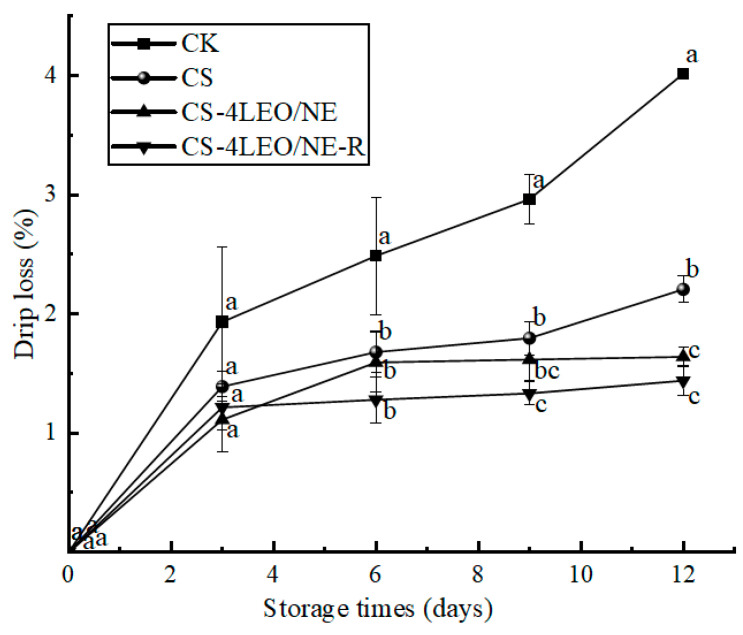
Changes in the drip loss rate of pork samples during different storage periods (different letters indicate statistically significant differences (*p* < 0.05) at same storage time).

Drip loss is an important indicator for evaluating pork quality [45]. Figure 9 shows that the drip loss rate of all groups tended to increase with increasing storage time. This may be attributed to the fact that microbial metabolic activities could disrupt the muscle tissue structure with longer storage times, causing an increase in drip loss [46]. Notably, the drip loss rate of the CS-4LEO/NE-R coating group was consistently lower than that of the other experimental groups throughout the whole storage period. This may be attributed to the fact that the coating had a better inhibitory effect on the microorganisms on the surface of the pork (as proved in Figure 5, Figure 6 and Figure 7). In addition, the thin film on the surface of the pork that was formed due to the coating treatment could also help to reduce the water loss of the pork.

#### 3.3.4. TVB-N

**Figure 10:**
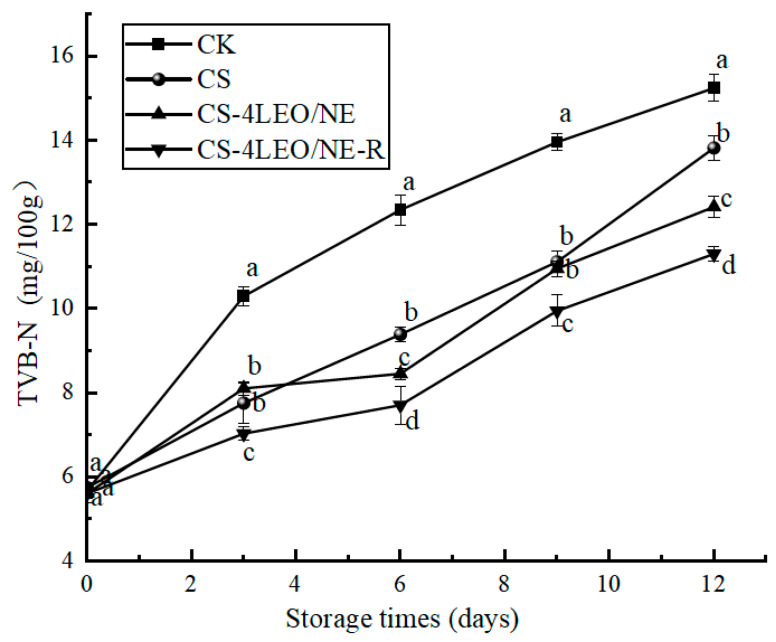
Changes in TVB-N of pork samples at different storage periods (different letters indicate statistically significant differences (*p* < 0.05) at same storage time).

TVB-N is an important indicator reflecting the degradation of proteins and amines in meat products [47]. As shown in Figure 10, the initial TVB-N values of all groups were lower than 6.00 mg/100 g, and the values tended to increase with increasing storage time. This was due to the decomposition of proteins and nitrogenous substances during the storage process [48]. The increase rate of TVB-N values in different groups had the following order: CK > CS > CS-4LEO/NE > CS-4LEO/NE-R. After 12 days of storage, the TVB-N value of the CK group reached 15.24 ± 0.32 mg/100 g, whereas the value of the CS-LEO/NE-R group was only 11.29 ± 0.18 mg/100 g. The results revealed that the CS-4LEO/NE-R coating treatment was the most effective in inhibiting microbial activities and delaying protein decomposition.

#### 3.3.5. TBARS

**Figure 11:**
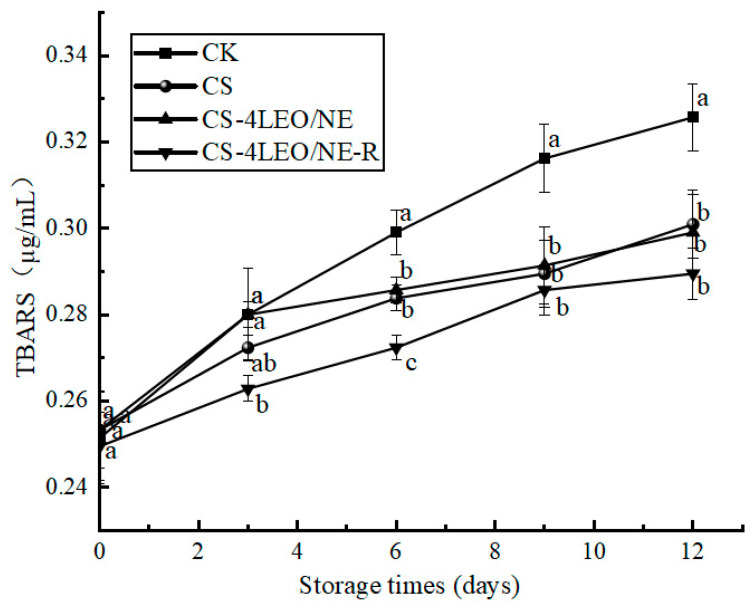
Changes in the TBARS of pork samples during different storage periods (different letters indicate statistically significant differences (*p* < 0.05) at same storage time).

Lipid oxidation is an important factor contributing to the spoilage of meat products, and it can be reflected by the TBARS value [49]. Figure 11 shows that the TBARS values of all groups tended to increase throughout the entire period of storage. The CK group shows the fastest increase rate, suggesting the highest level of lipid oxidation. On the 12th day, the TBARS values of the CS, CS-4LEO/NE and CS-4LEO/NE-R groups increased to 0.301 ± 0.008, 0.299 ± 0.009 and 0.290 ± 0.006 µg/mL, respectively. Notably, the TBARS values of the CS-4LEO/NE-R group were consistently lower than those of other groups during the whole storage, which might be related to the good antioxidant capacity of the coating, as proved in Figure 4.

#### 3.3.6. TVC

**Figure 12:**
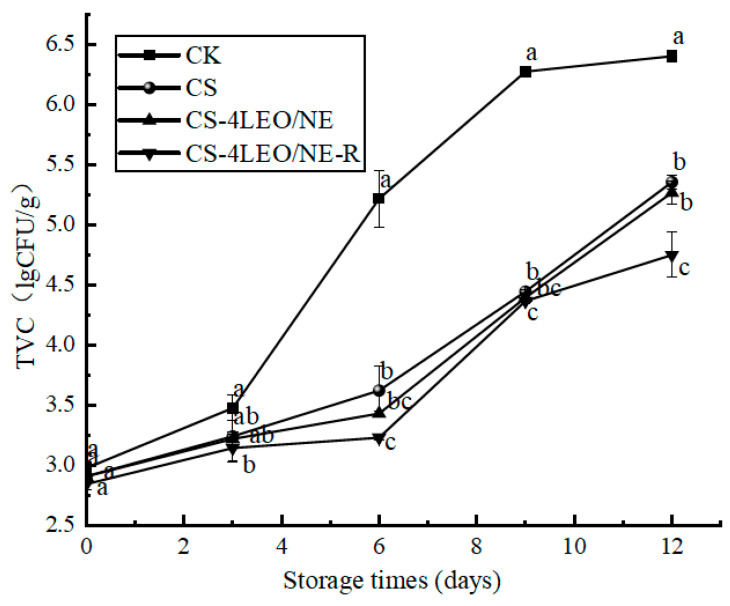
Changes in the TVC of pork samples at different storage periods (different letters indicate statistically significant differences (*p* < 0.05) at same storage time).

Figure 12 shows that the TVC values of all groups continued to increase during storage, but the increase rates of the coating treatment groups were significantly (*p* < 0.05) lower than the CK group. This may be mainly due to the antibacterial effects of the CS, CS-4LEO/NE and CS-4LEO/NE-R coatings, as proved in Figure 5, Figure 6 and Figure 7. The initial TVC values of all the samples were lower than 3.00 lg CFU/g. The TVC value of the CK group reached 6.28 ± 0.02 lg CFU/g after being stored for 9 d, which exceeded the standard (6 lg CFU/g) of Chinese national standard GB2707-2016 [50]. However, the TVC values of the CS, CS-4LEO/NE and CS-4LEO/NE-R groups were all lower than the limit value, even after 12 days of storage. Notably, the TVC value of the CS-4LEO/NE-R group was only 4.72 ± 0.19 lg CFU/g on the 12th day. The above results indicated that the CS-4LEO/NE-R coating treatment could effectively inhibit the growth and reproduction of microorganisms in pork, and thus extend its shelf-life.

## 4. Conclusions

In this study, CS was used as the coating matrix, and LEO-NE-R was used as a functional substance to prepare a coating for pork preservation. The introduction of rutin could enhance the slow-release performance of the nanoemulsion for LEO. The CS-based coating activated by LEO-NE-R showed stable film-forming properties. Its antioxidant and bacteriostatic activities were improved and positively correlated with the content of the incorporated nanoemulsion. The activated CS-based coating treatment could effectively slow the color deterioration, the increase in pH and the drip loss, while inhibiting the oxidation of lipids, the degradation of proteins and the growth and reproduction of microorganisms. These results can provide insight for the development of new and efficient meat preservation technologies.

## Figures and Tables

**Table 1:** Average particle size, PDI and zeta potential of the composite nanoemulsion.

Samples	MD (nm)	PDI	ZP (mV)
LEO-NE	239.67 ± 2.83 ^a^	0.28 ± 0.04 ^a^	−21.07 ± 0.06 ^b^
0.05 LEO-NE-R	175.50 ± 0.98 ^d^	0.21 ± 0.01 ^b^	−25.57 ± 0.85 ^a^
0.1 LEO-NE-R	178.93 ± 0.67 ^d^	0.22 ± 0.01 ^b^	−25.83 ± 0.31 ^a^
0.2 LEO-NE-R	199.37 ± 2.06 ^c^	0.23 ± 0.01 ^b^	−20.80 ± 0.26 ^b^
0.3 LEO-NE-R	233.63 ± 3.00 ^b^	0.23 ± 0.01 ^b^	−21.17 ± 0.40 ^b^

Note: Different letter superscripts in the same column indicate statistically significant differences (*p* < 0.05).

**Table 2:** The diameters of inhibition circle of different coatings against *S. aureus*, *L. monocytogenes* and *E. coli.*

Samples	Inhibition Zone Diameter (mm)		
	*S. aureus*	*L. monocytogenes*	*E. coli*
CS	14.29 ± 0.20 ^g^	12.88 ± 0.13 ^g^	12.51 ± 0.07 ^f^
CS-1LEO/NE	14.89 ± 0.53 ^f^	13.09 ± 0.14 ^g^	13.09 ± 0.14 ^e^
CS-2LEO/NE	14.90 ± 0.23 ^f^	13.64 ± 0.08 ^ef^	13.47 ± 0.10 ^cd^
CS-3LEO/NE	15.88 ± 0.05 ^cd^	13.77 ± 0.07 ^de^	13.44 ± 0.21 ^d^
CS-4LEO/NE	16.57 ± 0.08 ^ab^	14.10 ± 0.03 ^c^	13.81 ± 0.03 ^b^
CS-1LEO/NE-R	15.27 ± 0.12 ^ef^	13.45 ± 0.03 ^f^	13.29 ± 0.20 ^de^
CS-2LEO/NE-R	15.61 ± 0.16 ^e^	14.04 ± 0.22 ^cd^	13.76 ± 0.14 ^bc^
CS-3LEO/NE-R	16.19 ± 0.13 ^bc^	14.41 ± 0.14 ^b^	14.01 ± 0.10 ^b^
CS-4LEO/NE-R	16.74 ± 0.09 ^a^	15.27 ± 0.18 ^a^	14.40 ± 0.14 ^a^

Note: Different letter superscripts in the same column indicate statistically significant differences (*p* < 0.05).

**Table 3:** Changes in the color value of pork during different storage periods.

Parameter	Sample	Storage Times/d				
		0	3	6	9	12
L*	CK	50.33 ± 0.8 ^a^	50.40 ± 0.49 ^b^	50.22 ± 0.44 ^ab^	48.91 ± 1.68 ^a^	46.45 ± 0.66 ^c^
	CS	50.80 ± 1.9 ^a^	52.64 ± 0.63 ^a^	48.96 ± 1.16 ^b^	47.93 ± 1.45 ^a^	47.41 ± 0.72 ^bc^
	CS-LEO/NE	49.89 ± 0.5 ^a^	51.23 ± 0.17 ^b^	49.64 ± 0.35 ^ab^	49.45 ± 0.71 ^b^	48.31 ± 0.32 ^ab^
	CS-LEO/NE-R	50.28 ± 0.3 ^a^	52.97 ± 0.44 ^a^	50.73 ± 0.48 ^a^	49.62 ± 0.42 ^a^	48.95 ± 0.82 ^a^
a*	CK	9.40 ± 0.13 ^b^	9.15 ± 0.51 ^b^	6.77 ± 0.39 ^b^	5.93 ± 0.33 ^c^	5.13 ± 1.03 ^c^
	CS	8.55 ± 0.16 ^c^	8.60 ± 0.14 ^b^	7.40 ± 0.76 ^b^	6.13 ± 0.17 ^bc^	5.66 ± 0.17 ^bc^
	CS-LEO/NE	9.06 ± 0.06 ^b^	9.09 ± 0.34 ^b^	7.44 ± 0.73 ^b^	7.09 ± 0.82 ^b^	6.82 ± 0.28 ^b^
	CS-LEO/NE-R	9.93 ± 0.24 ^a^	10.42 ± 0.26 ^a^	9.19 ± 0.31 ^a^	9.09 ± 0.25 ^a^	9.24 ± 0.42 ^a^
b*	CK	5.54 ± 0.09 ^a^	6.10 ± 0.40 ^a^	7.53 ± 0.64 ^a^	7.56 ± 0.54 ^a^	8.30 ± 0.48 ^a^
	CS	5.33 ± 0.13 ^b^	5.94 ± 0.53 ^a^	6.75 ± 0.25 ^ab^	7.07 ± 0.33 ^ab^	7.65 ± 0.26 ^ab^
	CS-LEO/NE	5.23 ± 0.04 ^b^	5.33 ± 0.15 ^a^	6.09 ± 0.26 ^bc^	6.65 ± 0.71 ^ab^	7.15 ± 0.29 ^b^
	CS-LEO/NE-R	5.22 ± 0.04 ^b^	5.32 ± 0.73 ^a^	5.86 ± 0.11 ^c^	6.35 ± 0.22 ^b^	6.88 ± 0.51 ^b^

Note: Different letter superscripts in the same column indicate statistically significant differences (*p* < 0.05).

## Data Availability

The original contributions presented in this study are included in the article. Further inquiries can be directed to the corresponding author.
